# Understanding and Synergy: A Single Concept at Different Levels of Analysis?

**DOI:** 10.3389/fnsys.2021.735406

**Published:** 2021-11-18

**Authors:** Mark L. Latash

**Affiliations:** ^1^Department of Kinesiology, The Pennsylvania State University, University Park, PA, United States; ^2^Moscow Institute of Physics and Technology, Dolgoprudnyj, Russia

**Keywords:** referent coordinate, uncontrolled manifold, stability, motor equivalence, efference copy, iso-perceptual manifold

## Abstract

Biological systems differ from the inanimate world in their behaviors ranging from simple movements to coordinated purposeful actions by large groups of muscles, to perception of the world based on signals of different modalities, to cognitive acts, and to the role of self-imposed constraints such as laws of ethics. Respectively, depending on the behavior of interest, studies of biological objects based on laws of nature (physics) have to deal with different salient sets of variables and parameters. Understanding is a high-level concept, and its analysis has been linked to other high-level concepts such as “mental model” and “meaning”. Attempts to analyze understanding based on laws of nature are an example of the top-down approach. Studies of the neural control of movements represent an opposite, bottom-up approach, which starts at the interface with classical physics of the inanimate world and operates with traditional concepts such as forces, coordinates, etc. There are common features shared by the two approaches. In particular, both assume organizations of large groups of elements into task-specific groups, which can be described with only a handful of salient variables. Both assume optimality criteria that allow the emergence of families of solutions to typical tasks. Both assume predictive processes reflected in anticipatory adjustments to actions (motor and non-motor). Both recognize the importance of generating dynamically stable solutions. The recent progress in studies of the neural control of movements has led to a theory of hierarchical control with spatial referent coordinates for the effectors. This theory, in combination with the uncontrolled manifold hypothesis, allows quantifying the stability of actions with respect to salient variables. This approach has been used in the analysis of motor learning, changes in movements with typical and atypical development and with aging, and impaired actions by patients with various neurological disorders. It has been developed to address issues of kinesthetic perception. There seems to be hope that the two counter-directional approaches will meet and result in a single theoretical scheme encompassing biological phenomena from figuring out the best next move in a chess position to activating motor units appropriate for implementing that move on the chessboard.

## Introduction

Two terms, “understanding” (as used in cognitive neuroscience) and “synergy” (as used in movement neuroscience) seem to be closely related to each other. Indeed, *understanding* has been viewed as the discovery of co-variation between groups of relevant cognitive variables based on optimization, likely related to minimizing energy expenditure inside the system (Yufik, 2013, 2019). It has been also linked to one’s ability to transform multiple lower-level concepts into a unified higher-level concept, meaning (Perlovsky, 2016). Understanding leads to overcoming the inertia of prior learning and enabling the construction of adequate responses under novel and unfamiliar circumstances (Yufik and Friston, 2016).

The word *synergy* has been used in the field of motor control with two implied meanings: Grouping numerous elements into stable groups to reduce the number of variables manipulated by the brain and co-varying group involvement with the purpose to ensure dynamical stability of actions in the unpredictable environment (reviewed in Bernstein, 1947; Latash, 2008, 2020a,b). Optimization ideas have been used broadly to account for the observed grouping of elements and their time evolution during typical actions (reviewed in Prilutsky and Zatsiorsky, 2002; Diedrichsen et al., 2010). So, both notions can be viewed as combinations of grouping plus co-variation plus optimization. Can they represent fundamentally similar neural mechanisms reflecting different stages of the evolutionary process, from *synergies* seen across numerous species to *understanding* claimed to be unique to the human species (Yufik, 2019)?

The contrast between the two notions becomes obvious if one considers typical spaces of variables where these notions are defined and applied: The spaces of mental models and meanings in studies of understanding vs. the spaces of variables from classical physics such as forces and coordinates (and their derivatives) in studies of synergies. The two notions and the corresponding spaces reflect two classes of approaches to neuroscience problems based on laws of nature: top-down and bottom-up. The former tries to describe aspects of cognition, including the one of understanding. It starts with accepting a set of axiomatic notions such as the mental model and meaning. The second starts from the interface with the inanimate world and operates with notions from classical physics, in particular classical mechanics. Of course, top and bottom are defined within this classification relatively arbitrarily. For example, one can start from classical physics and chemistry or even physics of elementary particles, and consider the simplest motor actions as examples of top-down analysis.

This article follows the bottom-up approach as compared to typical studies of cognition. It starts with trying to identify terms within the biology-specific adequate language (Gelfand, 1991; Gelfand and Latash, 1998), missing in the physics of inanimate nature. This leads to two important concepts, those of parametric control and spatial referent coordinates (RCs) originating from the classical equilibrium-point hypothesis (Feldman, 1966, 1986, 2015). Further, the concept of synergy is linked to arguably the most important feature of biological actions, their controlled task-specific stability. The ideas of synergic control and hierarchical control with spatial RCs are merged naturally (Latash, 2010, 2019, 2021a) leading to the possibility of ensuring dynamic stability of actions at levels ranging from groups of motor units to the whole body. This is an actively developed field with applications to such areas as motor learning, neurological disorders, and rehabilitation.

Further, we try to expand this approach to the field of perception. This development faces major problems with experimental verification because salient variables are not as readily measurable objectively. Nevertheless, there are promising recent theoretical and experimental studies suggesting the existence of percept-stabilizing synergies. At the end of the article, we return to the notion of understanding and try to link it to the stage of discovery during motor skill acquisition.

## The Neural Control of Biological Action

Bernstein was arguably the first to emphasize that the brain could not in principle prescribe such peripheral variables as forces and trajectories given the typical time delays associated with processing and conduction of neural signals, and time-varying changes in the external forces and intrinsic body states, which can never be perfectly predicted in advance (Bernstein, 1947; translation in Latash, 2020b). According to one of the influential theories of motor control, this problem is solved by using parametric control: biological movements are produced by changing parameters within the relations between actively produced forces and coordinates (reviewed in Feldman, 2015; Latash, 2019). In physical terms, these parameters have been associated with spatial referent coordinates for the involved effectors. Their physiological meaning is threshold for muscle activation associated with subthreshold depolarization of corresponding neuronal pools.

An alternative approach to problems of motor control and coordination has been developed assuming that the brain performs computations (addressed as “internal models”, e.g., Wolpert et al., 1998; Kawato, 1999; Shadmehr and Wise, 2005) to plan, predict, and prescribe peripheral mechanical variables produced by muscles, joints, and other effectors. Major differences between this approach and the one following Bernstein’s traditions have been reviewed earlier (Ostry and Feldman, 2003; Feldman and Latash, 2005; Feldman, 2015). The purpose of this article is not to contribute to these polemics but to follow Bernstein’s definition and understanding of synergies and review recent studies exploring synergies at different levels and in different domains.

Within the classical equilibrium-point hypothesis for the control of a single muscle (Feldman, 1966, 1986), the salient parameter is the threshold (λ) of the stretch reflex expressed in units of muscle length and, simultaneously, representing subthreshold depolarization of the corresponding alpha-motoneuronal pool expressed in units salient for neurophysiological processes, millivolts. Changing λ can lead to various changes in peripheral variables such as muscle activation level, force (*F*), and length (*L*), depending on the external force field, in line with Bernstein’s insight.

The idea of control with spatial RCs has been generalized to both multi-muscle systems that take part in typical functional actions and to intra-muscle subsystems. Whole-body actions, for example, pointing, are assumed to be controlled with a relatively low-dimensional RC specified at the level of task-relevant effectors, for example, a three-dimensional coordinate during typical arm reaching or pointing actions. Further, there is a sequence of few-to-many transformations leading to higher-dimensional RCs at hierarchically lower levels such as joints and muscles. This process is associated with apparent problems of redundancy because a small number of constraints are used to specify a large number of variables. As discussed later, the classical formulation of this problem (Bernstein, 1947; Turvey, 1990) is misleading and has to be replaced with the concept of *abundance* (Latash, 2012), which is not a source of the computational problem but an evolutionary advantageous design that ensures both stability of actions and their flexibility, i.e., adjustment to the changing external conditions.

Recently, the idea of control with RCs has been expanded in the opposite direction, i.e., inside the muscle (Madarshahian et al., 2021). Indeed, a number of muscles in the human body are viewed as combinations of compartments (Jeneson et al., 1990; Mariappan et al., 2010), i.e., groups of motor units united by both functional and anatomical criteria. Each compartment consists of numerous motor units, which may be viewed as the smallest unit of control. A motor unit is controlled by a single alpha-motoneuron and, as such, it obeys the law “all or none”, which means that it can be recruited only as a whole. The contribution of a motor unit to muscle (or compartment) activation and mechanics can be varied by changing the frequency (*f*_MU_) of action potential generation by the corresponding alpha-motoneuron.

**Figure 1:**
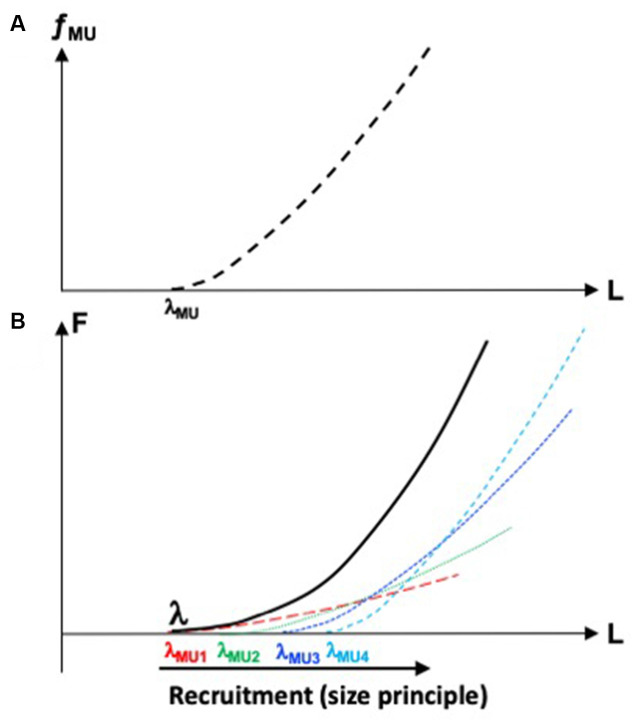
**(A)** The dependence between frequency of firing of a motor unit, *f*_MU_, and length of the group of muscle fibers forming this motor unit. Its threshold of activation is λ_MU_. **(B)** The muscle force-length, *F*(*L*) characteristic (solid line) may be viewed as a superposition of motor unit characteristics (dashed lines). The motor units are recruited in an orderly fashion, from smaller to larger ones.

Figure 1A illustrates the dependence between *f*_MU_ and the length of a group of muscle fibers forming the motor unit. It is characterized by the threshold of activation, λ_MU_ (motor units are typically recruited in an orderly fashion, from the smallest to the largest ones, Henneman et al., 1965) and the specific shape of the dependence of *f*_MU_ on muscle length. An increase in *f*_MU_ corresponds to an increase in the contribution of this particular motor unit to muscle force. Hence, the muscle *F*(*L*) characteristic may be viewed as a superposition of motor unit *f*_MU_(*L*) characteristics (Figure 1B). Of course, expansion of the control with RC into spaces of muscle compartments and motor units is associated with even more glaring problems of redundancy or, if one accepts the concept of abundance, with even more opportunities to ensure dynamical stability of salient task-related performance variables.

Recently, the idea of control with RCs has been developed to account for a variety of phenomena including effects of motor adaptation to unusual force fields (Gribble and Ostry, 2000), motor learning (Turpin et al., 2016), neuronal population coding of control variables by the brain (Feldman, 2019), agonist-antagonist coactivation (Latash, 2018a), perceptual errors (Latash, 2018b), and certain types of neurological disorders including spasticity (Jobin and Levin, 2000; Mullick et al., 2013). This approach is based on the solid foundation of experimental findings in studies ranging from those involving animal preparations (Feldman and Orlovsky, 1972; Hoffer and Andreassen, 1981) to healthy humans (Feldman, 1966; Schmidt and McGown, 1980; Latash and Gottlieb, 1990; Latash, 1992).

## Controlled Stability of Action

The concept of *synergy* in movement studies has been used at least since the XIXth century as a synonym of the word *coordination*; respectively, *asynergia* and *dyssynergia* have been used as synonyms of impaired coordination (Babinski, 1899). Bernstein incorporated this concept into his multi-level hierarchical scheme for the control of movements as the second from the bottom level. Its full name was “The level of synergies and patterns or the thalamo-pallidar level” emphasizing the importance of the loops through the basal ganglia, an insight supported by recent studies (reviewed in Latash and Huang, 2015). According to Bernstein, the level of synergies serves two main functions: (1) organizing numerous elements into groups; and (2) ensuring the dynamical stability of movements.

The former function of synergies is directly related to the famous problem of motor redundancy (Bernstein, 1947, 1967). Bernstein was arguably the first to pay attention to the fact that each natural movement involves numerous elements at multiple levels of analysis, kinetic, kinematic, muscle activation, etc. The number of elements is larger than the number of constraints associated with typical tasks and, therefore, an infinite number of solutions exist. In his main book, Bernstein (1947) was ambiguous with respect to this problem. In different sections, he emphasized both the elimination of redundant degrees-of-freedom considered as the main problem of motor control and benefits of having extra degrees-of-freedom. How does the central nervous system select specific solutions observed during movements? Bernstein’s expression “elimination of redundant degrees-of-freedom” as the method of finding unique solutions for typical problems of motor redundancy dominated the field until recently. In fact, the problem of motor redundancy has another component: Even for a single element, movement from an initial to a final state can proceed along an infinite number of trajectories. How does the central nervous system select specific trajectories from this set? So, there is a problem of state redundancy and a problem of trajectory redundancy. During natural movements, both problems coexist.

Arguably, the most commonly used method to solve such problems has been optimization formulated as search for a minimum (or maximum) of a cost function in different spaces of variables, mechanical, neurophysiological, and psychological (reviewed in Seif-Naraghi and Winters, 1990; Prilutsky and Zatsiorsky, 2002). Recently, methods of optimal feedback control have been used to find solutions for such problems (Todorov and Jordan, 2002; Diedrichsen et al., 2010). There are two obvious problems with most such methods. First, they assume that the neural controller computes cost function values, typically based on performance variables, over movement time prior to movement initiation, i.e., that movement time is known in advance and time profiles of the relevant variables can be accurately predicted over the future movement. Second, the choice of the cost function is usually rather arbitrary, reflecting personal theoretical preferences.

The ill-posed nature of the problem of motor redundancy can be illustrated with the example of excessive muscle co-activation seen at early stages of skill acquisition (Bernstein, 1947). Bernstein viewed this phenomenon as an attempt to mitigate the problem of redundancy by limiting the kinematic space of possible movements. This may be true if the problem is considered at the level of joint kinematics. However, co-activation obviously makes the problem worse at the level of muscle activation and motor unit recruitment. This example suggests that, before the problem is solved, it has to be clearly formulated at the level of neural control variables, such as RCs, not peripheral mechanical variables.

Recently, the problem of motor redundancy has been reformulated as the *principle of abundance* (Gelfand and Latash, 1998; Latash, 2012). This reformulation emphasizes the importance of variability in both neural and motor processes and postulates that the brain facilitates “good enough” solutions (Loeb, 2012; Akulin et al., 2019) and uses the abundance of elements to ensure desired dynamical stability of those solutions with respect to salient performance variables. The idea of abundance follows naturally the classical Bernstein’s study of hammering by professional blacksmiths (Bernstein, 1930) where he showed that the trajectory of the tip of the hammer showed less inter-trial variability compared to the trajectories of individual joints. The importance of motor variability has also been illustrated by pathologies characterized by unusually low variability (e.g., low postural sway in advanced-stage Parkinson’s disease, Horak et al., 1992) and the links between low variability and incidence of chronic pain in healthy persons (Madeleine et al., 2008; Madeleine and Madsen, 2009).

The principle of abundance fits well the aforementioned definition of the level of synergies in the multi-level hierarchical control scheme by Bernstein (1947) and Latash (2020a), in particular its assumed role in ensuring dynamical stability of actions. This approach is tightly linked to the concept of *uncontrolled manifold* (UCM; Schöner, 1995; Scholz and Schöner, 1999). According to the UCM-hypothesis, the central nervous systems acts in multi-dimensional spaces of elemental variables and structures variance in those spaces to allow relatively large variance along a subspace where a salient performance variable does not change (the UCM for that variable) while minimizing variance leading to changes in that variable, i.e., in the orthogonal to the UCM space (ORT space).

**Figure 2:**
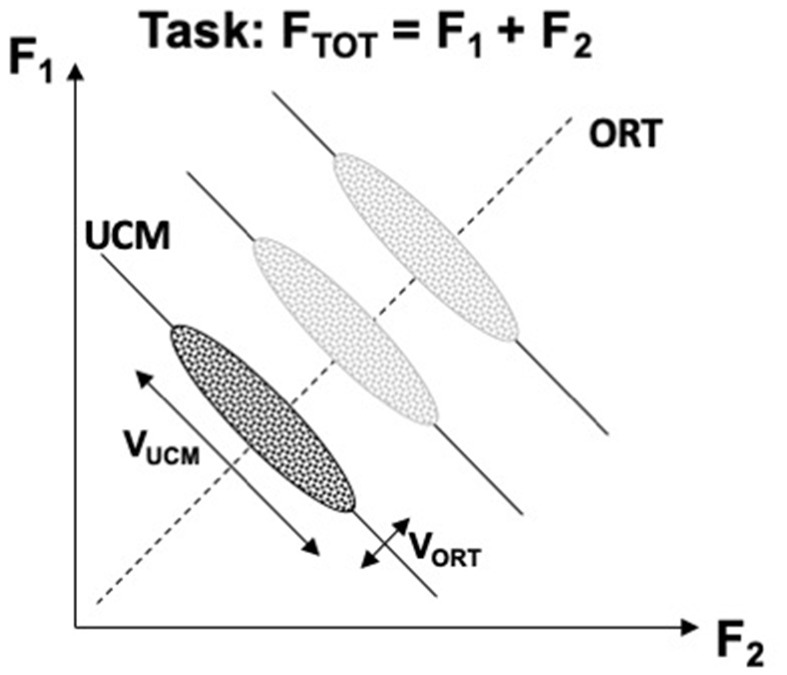
An illustration of the UCM concept for the task of producing constant total force (*F*_TOT_) while pressing with two fingers. A cloud of data points across trials is shown with an ellipse. Note that variance along the solution space (uncontrolled manifold, UCM, solid lines) is larger than orthogonal to the UCM (ORT, dashed line), *V*_UCM_ > *V*_ORT_. Producing different force magnitudes is associated with UCM shifts, while the relative location and shape of the data clouds remain about the same (lighter images). *V*_UCM_, variance within the UCM; *V*_ORT_, variance within the ORT.

Figure 2 illustrates the UCM concept for the task of producing constant total force (*F*_TOT_) while pressing with two independent effectors, e.g., two fingers. The inter-trial data cloud is expected to form an ellipse elongated along the UCM. Quantifying variance per dimension within the UCM and within the ORT is expected to produce an inequality *V*_UCM_ > *V*_ORT_ if indeed the central nervous system stabilizes the potentially important performance variable (*F*_TOT_, in this example) at the expense of other variables produced by the same set of effectors. If the subject in this experiment is asked to produce a different force magnitude, the UCM shifts, but the location and shape of the data cloud are expected to be robust (as illustrated for three *F*_TOT_ magnitudes in Figure 2). It has been suggested that the location of the center of the inter-trial cloud may reflect an optimization principle, whereas the shape of the cloud reflects the stability of the performance variable (Park et al., 2010). Assuming that there exists a single optimal solution and any deviations from this solution incur extra costs, large *V*_UCM_ (reflecting high stability) implies large deviations from the center of the data point distributions, i.e., large violations of the optimality principle.

Large magnitudes of *V*_UCM_ are reflections of low stability along the UCM, which is functionally important. Indeed, large *V*_UCM_ allows performing secondary tasks with the same set of elements without negative interference with the original task. In addition, low stability along the UCM channels effects of unexpected perturbations into the UCM thus protecting the salient variable from such perturbations. For example, imagine walking along the beach while carrying in the dominant hand a mug filled with hot coffee. At the level of kinematics, vertical mug orientation is a salient performance variable, which gets contributions from numerous kinematic variables—joint angles along the body and the arm. During walking, unexpected perturbations emerge frequently, e.g., when stepping on a pebble, unexpected surface, etc. A multi-joint synergy stabilizing the mug orientation helps channel the kinematic effects of such perturbations into the respective UCM. You can lean and pick up a shell without spilling the coffee, which requires using a subset of joints of the body; this can be done by limiting joint rotations to the UCM. Clinical studies have confirmed the importance of high *V*_UCM_ magnitudes by showing that low indices of stability seen in certain groups of neurological patients are associated primarily with low magnitudes of *V*_UCM_, not with large magnitudes of *V*_ORT_ (Falaki et al., 2017; Jo et al., 2017).

A number of schemes have been suggested leading to the typical structure of variance for stabilized performance variables (*V*_UCM_ > *V*_ORT_). These include short-latency feedback loops within the central nervous system, somewhat similar to the classical system of recurrent inhibitions, as well as feedback projections from peripheral sensory endings (Latash et al., 2005; Martin et al., 2009). Similar clouds of data points elongated along the solutions space have been reported in modeling studies based on the minimal intervention principle (Todorov and Jordan, 2002) and implemented using optimal feedback control schemes (reviewed in Diedrichsen et al., 2010). Within those schemes, deviations in spaces of elemental variables are corrected by the central nervous system only if they introduce errors into salient performance variables.

**Figure 3:**
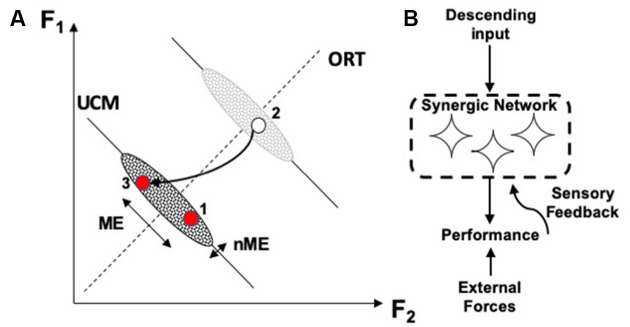
**(A)** A perturbation leads to a deviation of the system from an initial point 1 to point 2. During quick corrective action (to point 3), motor equivalent (ME) deviations along the corresponding UCM are larger than non-ME deviations. **(B)** Large ME deviations are expected if corrective signals serve as perturbations into the neural network ensuring the corresponding synergy.

The different stability along the UCM and along ORT leads to a particular signature of the phenomenon of motor equivalence. If a person is instructed to correct an ongoing action in cases of perturbations affecting a salient performance variable, corrections show very large motor equivalent components, i.e., deviations along the corresponding UCM (Figure 3; Mattos et al., 2011, 2015). In other words, deviations of elemental variables during the corrections show large components that do not correct anything, i.e., they are wasteful from the point of view of energy expenditure. Such large motor equivalent deviations are expected if corrective signals generated by the brain are seen as inputs (perturbations) into a neural network forming the corresponding synergy (Figure 3). Studies of motor equivalent and non-motor equivalent deviations have confirmed their relationship to the *V*_UCM_ and *V*_ORT_ indices expected from statistics of folded distributions (Falaki et al., 2017).

Recently, the notion of performance-stabilizing synergies has been developed for spaces of hypothetical control variables, i.e., RCs at different levels of the presumed control hierarchy (Reschechtko and Latash, 2017, 2018; Reschechtko et al., 2018; Latash, 2021a). Indeed, the abundance of RCs at any control level allows (but does not dictate!) synergies stabilizing performance. Such synergies have been confirmed in multi-finger force production tasks (Ambike et al., 2016a,b, 2018).

Important findings in studies of motor synergies include the following (reviewed in Latash, 2008, 2019)

- The central nervous system can use a set of elemental variables to stabilize various performance variables in a task-specific manner;
- Synergies can be attenuated in anticipation of an action that requires a quick change in the salient performance variable. These phenomena have been addressed as anticipatory synergy adjustments;
- Unintentional drifts in performance are associated with loss of stability, which can be quantified in spaces of mechanical elemental variables and control variables; and
- Controlled stability suffers with advanced age, atypical development, and a range of neurological disorders. It can be improved with specialized training.

## The Origin of Stable and Illusory Percepts

Perception of one’s own body configuration, movements, and forces at the interface with the environment has been traditionally addressed as kinesthetic perception. Kinesthetic perception can be viewed as the process of measurement of salient variables and reporting them to oneself or others. The importance of both the efferent (motor related) and the afferent (sensory, generated at the periphery) signals for kinesthetic perception has been accepted for a long time, at least from the middle of the last century when Von Holst and Mittelstaedt (1950/1973) introduced the notion of *efference copy*, close in spirit to the notion of *corollary discharge* (Sperry, 1950). In the original formulation, the concept of efference copy was associated with a copy of signals sent by alpha-motoneurons to muscles. This signal was used to predict changes in afferent signals from proprioceptors induced by the future movement (so-called, *reafference*). Reafference was expected to interact with efference copy and produce reflex changes in movements only if it differed from the efference copy-based prediction. This understanding of efference copy has been criticized recently (Feldman, 2009, 2016) because it cannot explain how muscles can be relaxed after a movement to a new posture. Indeed, if muscles are relaxed efference copy is the same (zero) in both states, and any changes in afferent signals cannot be predicted based on efference copy changes. Hence, they have to produce reflex muscle activation in contrast to everyday observations.

**Figure 4:**
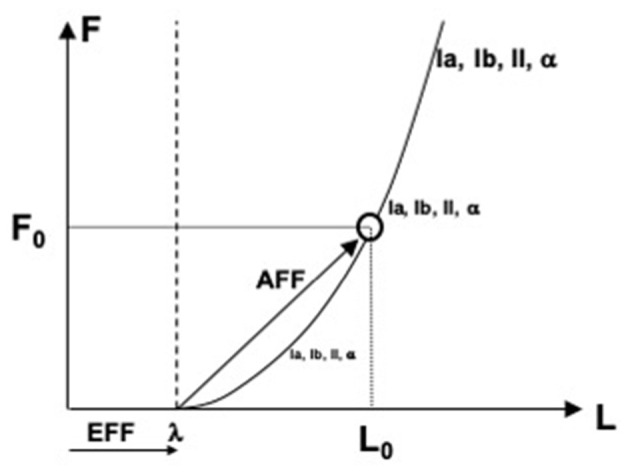
An illustration of perceiving muscle length (L) and force (F). Command to the muscle specifies the threshold of its stretch reflex (λ), which plays the role of referent coordinate. Many sensory and motor signals increase with deviation from λ along the force-length characteristic. Any of these signals may serve as afferent components of perceiving both force and length, F_0_ and L_0_.

In more recent studies, the role of the efferent process in perception has been associated with specifying a reference point (RC, see earlier). Indeed, to measure a physical variable, one has to have a reference point (from where to measure) and a tool (e.g., a ruler to measure distance). The efferent process has been assumed to supply the former component, and signals from peripheral receptors—the latter component (reviewed in Feldman, 2015, 2016; Latash, 2019, 2021b). Figure 4 illustrates the process of perceiving muscle length and force. Command to the muscle specifies the threshold of its stretch reflex (λ), which plays the role of RC. Many sensory signals show non-zero levels of activity when muscle length is shorter than λ and increase their activity level with deviation from λ along the force-length characteristic. These involve signals from length-sensitive and force-sensitive receptors as well as signals generated by the alpha-motoneurons innervating the muscle. Taken together, these signals form an abundant set, which may be viewed as the basis for stable kinesthetic percepts.

Imagine that you press with a hand against a stop such that no movement occurs. During changes in the pressing force, we have a veridical, undisturbed perception of steady posture. Where does this percept come from? Indeed, all signals from relevant peripheral receptors change. Signals from muscle spindles change with unavoidable changes in muscle fiber length (coupled to tendon length changes, such that the “tendon plus muscle” complex stays at the same length) and also changes in the activity of gamma-motoneurons, which change the sensitivity of spindle endings. Note that gamma-motoneurons change their activation level in parallel to the signals from alpha-motoneurons. There will be obvious changes in signals from force-sensitive Golgi tendon organs and from articular receptors, which are sensitive to the articular capsule tension. All the efferent signals will change as well. There seem to be no signals that are kept unchanged to correspond to the undisturbed perception of arm configuration. This observation has been interpreted as a reflection of all the signals, afferent and efferent, being constrained to a sub-space in the combined multi-dimensional afferent-efferent space—the iso-perceptual manifold (Latash, 2018b).

**Figure 5:**
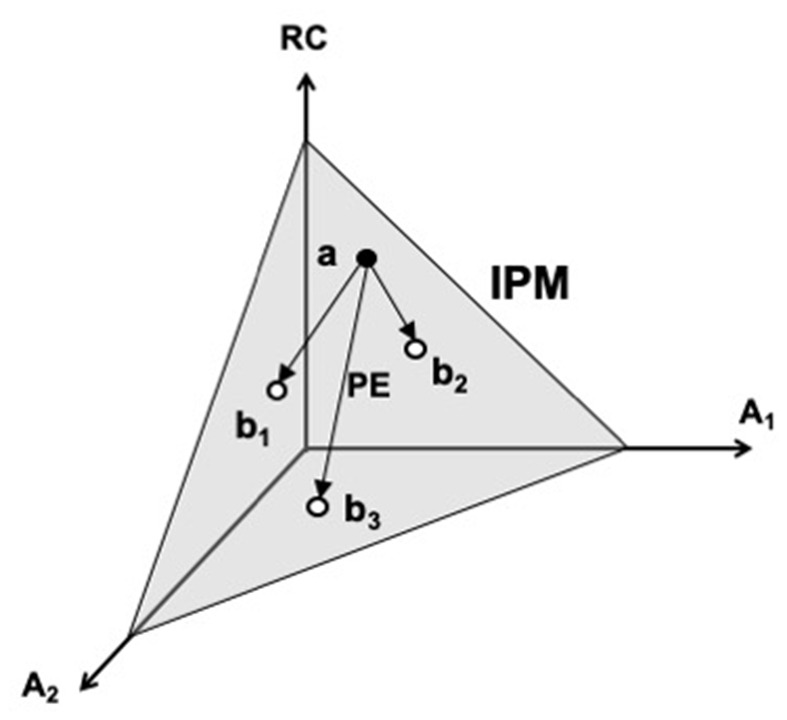
A cartoon illustration of the iso-perceptual manifold (IPM). One coordinate corresponds to an efferent signal (RC), and the other two—to two afferent signals (A_1_ and A_2_). Note that variations of all signals are possible within the IPM (e.g., from point *a* to points *b*_1_, *b*_2_, and *b*_3_) leading to the undisturbed perception of the salient variable. Such motion is perceptually-equivalent (PE). RC, referent coordinate.

A cartoon illustration of the iso-perceptual manifold in a three-dimensional space is shown in Figure 5. One coordinate corresponds to an efferent signal (RC), and the other two—to two afferent signals (A_1_ and A_2_). Note that variations of all signals are possible within the iso-perceptual manifold leading to the undisturbed perception of the salient variable. Such motion can be termed *perceptually-equivalent*, similarly to the motor equivalent motion described earlier. When the signals go outside the iso-perceptual manifold, perception of a change in the respective variable is reported, even if it is kept unchanged. The concept of the iso-perceptual manifold can be viewed as a definition of a stable kinesthetic percept. Indeed, there is no other definition addressing perceptual stability, which is a functionally very important feature of perception, crucial in the evolutionary process.

The iso-perceptual manifold concept implies that accurate perception of a functionally important variable can be associated with variable efferent and afferent signals to and from the involved elements. As a result, perception of variables produced by the elements may be less accurate when they participate in a multi-element action compared to their perception in single-element actions and to the perception of a variable produced by all the elements together. This prediction has been confirmed experimentally showing that perception of finger force is more precise and less variable during single-finger force production tasks as compared to multi-finger tasks (Cuadra and Latash, 2019; Cuadra et al., 2021b).

The described scheme can account for kinesthetic illusions, in particular those induced by muscle vibration (Goodwin et al., 1972; Roll and Vedel, 1982; Lackner and Taublieb, 1984), a powerful stimulus for signals from velocity-sensitive sensory endings in muscle spindles (Brown et al., 1967; Matthews and Stein, 1969). Note that this scheme links the perception of kinematic and kinetic variables and predicts vibration-induced illusions of both position and force—a prediction confirmed experimentally (Cafarelli and Kostka, 1981; Reschechtko et al., 2018). Some of the most recent studies explored the potential role of changes in efference copy in kinesthetic illusions, in particular those seen during misperception of force following voluntary muscle coactivation and the drifts in force after turning the visual feedback off (Cuadra et al., 2020, 2021a; Latash, 2021b). Under those conditions, relatively large-amplitude force changes are either not perceived or even perceived as happening in the opposite direction. The authors interpreted those observations as reflections of using distorted efference copy signals. In other words, efference copy is not necessarily a copy of the ongoing efferent process, as suggested earlier based on observations of vibration-induced kinesthetic illusions (Feldman and Latash, 1982).

Some of the mentioned studies also reported differences between two methods used to report percepts: Using verbal reports based on a psychophysical scale and using the contralateral effector to match the perceived variable. Both methods may be seen as suboptimal for obvious reasons such as subjectivity, possible drifts in memorized scales, asymmetry of the effectors on the two sides of the body, and other factors. Those studies observed qualitative differences in the reported percepts based on the two methods (Cuadra et al., 2020, 2021b). For example, coactivating muscles under the instruction to keep the pressing finger force constant leads to an unintentional force increase by about 50%. When asked to report the force change verbally, the subjects report that the force dropped by a small magnitude. In contrast, when asked to match the force with the contralateral hand, the subjects overshoot the actually increased force (Cuadra et al., 2020).

These observations suggest that perceiving-to-report and perceiving-to-act may involve different neural circuits. This conclusion matches well the classical notions of dorsal and ventral brain streams introduced for visual perception (Goodale et al., 1991; Goodale and Milner, 1992; Kravitz et al., 2011). It generalizes these notions to proprioception (see also Proffitt et al., 2003; Zadra et al., 2016) with a possibility that this rule applies to other modalities as well.

## Elements of Philosophy of Biological Action

The development of the idea of control with spatial RCs to perception is promising. However, this bottom-up approach may hit serious obstacles when dealing with issues that have traditionally been considered as those of cognition. An attempt to couple cognitive problems, such as, for example, selecting a target for movement, has been made by Gregor Schöner and colleagues in the form of the neural field theory incorporated into a general framework involved in the generation of functional actions, which involves the control with spatial referent coordinates and the synergic control of movements (Erlhagen and Schöner, 2002; Martin et al., 2009, 2019). However, even this most advanced scheme is rather far from dealing with such concepts as *understanding*.

It is possible that another qualitative step is needed to move from the control of biological movements with spatial RCs (and related perceptual phenomena) to issues such as understanding the relations among objects and using this understanding for selection of future motor and non-motor actions, including cognitive actions. This problem seems to be directly related to finding sets of adequate variables for each new level of analysis where variables and methods developed to describe processes at other levels fail (cf. Gelfand, 1991). This problem is also related to the ideas developed by the French philosopher, Merleau-Ponty (1942/1963), of different levels of complexity and associated problems pertaining to processes in inanimate nature (“physical order”), biological systems (“life order”), and conscious systems (“human order”).

The theory of control of biological movements with spatial referent coordinates makes a step from laws of nature of the inanimate world to possible laws of nature involved in the motor function of living systems. Can the same basic notions and laws be applied to problems of psychology and cognition? Or, to approach the problem of interface between biological action and cognition from the other side, does the concept of *understanding* apply equally to the fields of animal (including human) movements and to cognitive tasks such as selecting an optimal move in the chess game? Do children *understand* how to use the hand to turn the doorknob when they learn to open the door?

Nikolai Bernstein would probably agree that *understanding* is related to creating a *synergy* within the relevant space of elemental variables although this requires expanding the concept of synergy beyond its definition in his hierarchical scheme for the control of actions (Bernstein, 1947; translation in Latash, 2020b). Within that scheme, Bernstein placed synergy at the second from the bottom level (Level B). Within the same scheme, the concept of understanding (not used in the book) seems to be applicable only at the two top levels, the Level of Actions (Level D) and the Level of Symbolic Actions (Level E). The differences within the hierarchical scheme are one of the factors that make using two words justifiable. So far, synergy has been linked to action stability but not to selecting targets for action or other decision-making steps. In contrast, the concept of understanding has been developed within a computational approach based on the idea of active inference linked to minimization of variational free energy for a variety of brain functions including the control of movement and decision-making (Friston, 2012; Friston et al., 2013, 2017).

There are several features that are shared by the concepts of synergy and understanding. Both involve organizing the elemental variables into a few basic groups (addressed in movement studies with many names including modes, modules, factors, and primitives, reviewed in Latash, 2020a). Both involve ensuring the stability of task-specific outcomes, which may be picking up a glass with water and moving it to the mouth or finding an optimal move winning the chess game. Indeed, the concept of stability seems highly relevant to understanding: unstable understanding is doubt, which can be equated to the development of or transition toward understanding.

In his most comprehensive book, Bernstein (1947) emphasized the feeling of discovery when learning a skill, which he associated with delegating the responsibility for certain features of the task to lower levels of control (he addressed them as “background levels”), which are typically not perceived consciously. Such discoveries were associated, in particular, with finding dynamically stable trajectories solving the task, i.e., using pre-existing or creating new synergies stabilizing salient variables. For example, after one learns how to ride a bicycle, it is not necessary to think about not falling down, and the brain can become preoccupied with other tasks (e.g., where to ride it to and for what purpose, or even reciting poetry) as long as the road does not present perturbations exceeding the range of dynamical stability.

Using a similar language, *understanding* is also equivalent to delegating certain groups of problems to lower levels such that one is able to take for granted solutions for those problems and to have time and energy to deal with something more exciting and challenging. Can one develop a computational toolbox to measure the ability to understand that could be equivalent to the toolbox associated with the UCM hypothesis described earlier? This would require defining sets of elemental variables, salient higher-level variables, and the mapping rules between the two. A better understanding would imply using broadly varying combinations of elemental variables resulting in acceptable solutions for the cognitive task at hand. Is there an inherent trade-off between understanding (in terms of ensuring the stability of task-solving processes) and optimization (e.g., in terms of energy, Yufik, 2019) similar to the one described earlier for movements (Park et al., 2010)? These are exciting questions without answers so far.

## Author Contributions

The author is responsible for conceiving the manuscript, analysis, and writing the final draft. The author is responsible for all aspects of work on this article.

## Conflict of Interest

The author declares that the research was conducted in the absence of any commercial or financial relationships that could be construed as a potential conflict of interest.

## Publisher’s Note

All claims expressed in this article are solely those of the authors and do not necessarily represent those of their affiliated organizations, or those of the publisher, the editors and the reviewers. Any product that may be evaluated in this article, or claim that may be made by its manufacturer, is not guaranteed or endorsed by the publisher.

## Abbreviations

*f*: function
F: force
L: length
λ: threshold of the stretch reflex
MU: motor unit
ORT: space orthogonal to the uncontrolled manifold
RC: referent coordinate
UCM: uncontrolled manifold
*V*_UCM_ and *V*_ORT_: variance within the UCM and within ORT.
